# Discussions in the Western New York Dental Association, at Its Fifth Annual Meeting, Held in the City of Lockport

**Published:** 1868-01

**Authors:** 


### Proceedings of Societies.

### DISCUSSIONS IN THE WESTERN NEW YORK DENTAL ASSOCIATION, AT ITS FIFTH ANNUAL MEETING, HELD IN THE CITY OF LOCKPORT.

An Essay upon the manner of preparing teeth for filling; and annealing gold for filling was read by Dr. A. P. Southwick.

Dr. J. Taft remarked, that he regarded it preferable to effect immediate separation of the teeth for filling proximal cavities, by wedges; finds less liability to periostitis than when separation is gradually effected by pressure; the immediate separation is in the aggregate less painful and annoying to the patient than gradual pressure; there is far less danger of injury to the tooth or surrounding parts. He had never had a case of devitalization of a tooth resulting from immediate separation. He uses a wedge between the necks of the teeth, with an assistant wedge if need be between the points of the teeth, driving them with the mallet alternately till the required space is obtained, and then driving the former in firmly and removing the latter.

He remarked that Dr. Palmer uses a third very thin wedge at the neck of the tooth as a protection to the gum and a guide for the separating wedge. When the teeth are very firmly set or crowded, he drives the wedges gradually, in some cases occupying fifteen minutes in effecting sufficient separation. Never knew a case of periostitis from immediate separation. Has even known it to allay the periostitis; sometimes can obtain sufficient space without wedging, by the necessary trimming down the thin friable walls of the cavity. Commonly mortises down into the proximal cavities of the molars from the crown surface of the tooth, thus allowing of direct pressure with the axis of the teeth upon the gold in filling. Thinks forming a V shaped space between the teeth by means of a file, ordinarily a reprehensible practice. By mortising down into the cavity, the filling is made perfect on the cervical wall where they are most liable to fail. The tooth is more easily filled too, as the cavity is perpendicular to the axis of the tooth.

The reason for annealing gold is not altogether to drive off the accumulation of moisture and dust. A more important reason is that annealing causes a change in the arrangement of the ultimate particles of the gold favorable to welding. Had experimented much with crystal gold, and has seen the changes in the arrangement of the crystals.

Dr. Daboll thinks that another reason for annealing gold is the expansion consequent upon heating, which allows a more intimate interlacing of its particles.

Prof. Taft admits that there is much in this, but gold after annealing loses its cohesiveness slowly, which with other reasons leads him to the conclusion that there is a change in the arrangement of its ultimate particles.

Dr. Barrett is a believer in the theory of the regularity of the form and arrangement of the ultimate particles of all matter. This regularity of arrangement around one common axis he calls for want of a better term, polarity of particles. This is a law that obtains in the crystalization of all atoms. Cited in support of this the well known fact of the polarization or regular arrangement of the particles of water, when crystalizing into ice. It is upon this theory that the specific gravity of ice is less than that of water.

An Essay upon the different methods of preparing gold and their relative merits, was read by Dr. Daboll.

In the discussion upon this subject, Dr. Whitney said that he obtained the best results in his practice by means of pellets freshly annealed, their size depending upon the size of the cavity to be filled; but is careful not to make them so thick or large but that they may be thoroughly condensed. He found a great deal of difference in the quality of gold foil even from the same makers.

Pending the discussion the following resolutions were offered by Dr. Southwick, and was unanimously adopted.

*Resolved*, That we, as Dentists, condemn the practice of gold-beaters putting up their foils in the name of other parties or dealers than the original beaters, and that in all cases for our own protection it is important that we know whose make of foil we are using, and that we recommend that members of this Association neither buy nor use gold foil that has not the name of the manufacturer plainly stamped upon the book.

*Resolved*, That the action of some of our prominent gold-beaters in refusing to put up their foils in other than in their own books and under their own names is commendable, and that they in preference to others should be sustained by the profession.

The Secretary was instructed to forward to each of the gold-beaters in the United States a copy of these resolutions.

Dr. Whitney proceeding said he thought there was more in this matter than appears at first sight, for we paid parties advertising foil as their own which is made by beaters whose foil is also in the market under their own name. Thought that if beaters could only send out foil at the risk of their own reputation, they would perhaps put foil that was not what it should be back in the crucible. They would, however, not care so much about jeopardizing the reputation of others. But Dentists buy foil, some of which proves to be bad, and unless they know who the maker is, they know not how to avoid him in the future.

Dr. Bristol thinks that gold-beaters cannot always tell whether their foil be good or bad till Dentists try it. Thinks too that the fault may frequently be found in our own defective manipulation, rather than in want of care or skill on the part of the manufacturer.

Dr. Daboll says that if a man devotes his whole time and attention to one branch of business, he ought to know it well enough to be aware of what kind of an article he sends out.

Prof. Taft thinks that the stamping of gold with other than the maker's name, is only a form of advertising for the parties. But such resolutions as these call the Dentist's attention to irregularities and thereby accomplish good. But manufacturers have difficulties to contend with that we do not realize. We should not censure them too much. In chemical processes with gold, electrical and other influences operate to vary the result, sometimes in one direction, at others in another.

After some other desultory remarks by different members, the Association listened to the reading of a thoroughly digested essay upon the best means of preventing the destruction of the deciduous teeth, by Dr. Frank French.

Dr. Whitney is aware that he has been dubbed Dr. Phosphate by his professional brethren, for his persistency in recommending those agents. He was glad to see the turn that Dr. French's essay has taken. The business must be commenced months before the patient can come into our hands. The preservation of the deciduous teeth rests very much with the mother while the patient is yet in embryo.

Prof. Taft is very much pleased with the tone of this paper. But bricks and mortar are not all that is needed to build a house. There must be assimilating and upbuilding power. The phosphates must be contained in a medium that will perfectly dissolve them. Then to assimilate them, there must be the proper forces ready; vitality now steps in, as the dominant power, and employs chemical and mechanical forces, and so overrules and directs them as that assimilation and structural development shall continually go on. The particles of matter that enter into the composition of the human system, are the bricks and mortar. The nervous system, the blood vessels, the absorbents, and the numberless other parts of the animal mechanism, are the channels and vehicles by which the materials are conveyed where they are wanted; but *vitality* is the great architect, who guides, governs, and directs the distribution of the integral parts, in the building up of that great temple—the human body.

The teeth should be used upon such food as will give them proper strength, as soon as they appear. Any part of the human body will deteriorate with want of exercise. The practice of feeding infants upon food that does not try the strength of their teeth, is destructive to those organs. In adults, where only one side of the mouth is used in mastication, the unused teeth are seldom in a healthy state. Make the child use both sides of the mouth in mastication. If we find a green stain upon the necks of the teeth, it is an indication that they are not sufficiently exercised; this would, by the attrition in mastication of solid food be worn away. Another reason for using more solid food for young children is, that the salivary glands operate better when the teeth are thus exercised.

Dr. Whitney has often noticed that during gestation, the teeth of the mother are often drawn upon to furnish proper aliment to those of the fœtus; even to their partial disintegration.

Prof. Taft said that this was very often the case, that the system of the mother is drawn upon to its injury, to supply her offspring. He had often fancied that at such times, the system laughingly took up, and gladly assimilated the phosphates that it was longing—pining for. If they be not supplied from outside, the assimilating processes in behalf of the embryo search them out in the system of the mother, and rob her to supply the want, for they *must* be procured somewhere.

Had some experience in giving phosphates to weak infants, suffering from difficult dentition, and usually there is a marked change for the better. After giving the preparation in some cases for but a few days, the teeth were erupted and the trouble ended. Irregularities and imperfect teeth, are often owing to a lack of phosphates in the system.

Dr. Chittenden had noticed that the Scotch, whose chief article of food is oatmeal, containing large quantities of phosphate of lime, seldom have imperfect teeth.

At the suggestion of several members miscellaneous business was now taken up.

Dr. Whitney spoke of protective legislation, and invited Prof. Taft to relate the means taken by Ohio Dentists to compass their end; which he did, giving a concise history of the movement in Ohio.

WEDNESDAY, A. M. SESSION.

Drs. McGill of Erie, and Blair of Columbus, Pa., were introduced, and by vote of the Association were invited to take part in the discussion.

An Essay upon Mechanical Dentistry was read by Dr. Barrett, followed by Dr. Cook upon the same subject.

It was asserted by the essayist, that owing to unequal contraction in cooling down the rubber work after vulcanizing, and to other causes some only partially understood, there was frequently after finishing up the plate, a spontaneous springing or warping, which injured the adaptation to the mouth. In sectional gum teeth this tendency was resisted in the anterior portion of the plate by the sectional gum blocks, as also by the arch of the plate, throwing the whole deflection upon the posterior portion. The remedy suggessted was, to save the original cast after vulcanizing, dry it out, and after becoming satisfied that the plate had sprung, place the plate carefully back upon the cast, and after slowly heating with a spirit lamp, spring it to its place.

Dr. Bristol never preserved his casts; never had any difficulty with the springing of plates, that he knew of; but had never had his attention called particularly to the matter. Never uses a vacuum chamber, but trims off the cast along the medial line. Trims off the cast a little also in the neighborhood of where the bicuspids are situated.

To prevent the spreading of joints in sectional teeth, he works the plaster about the teeth until it has set. This makes it very hard.

Dr. Requa gives time for plaster thoroughly to set to prevent spreading of the joints. If possible, allows it to stand over night, before opening the flask. For relieving the pressure in the palatine arch, cuts out from one to three thicknesses of tin foil, and lays it over the heel of the cast in the palatine arch.

Dr. Daboll has lately saved his casts, but cannot say that he has had trouble with the springing of plates.

Dr. Hayes has been obliged to spring two-thirds of his plates, before he considers them fit for wear.

Dr. Southwick has demonstrated by repeated experiments that rubber plates spring. The higher the palatine arch the more the plate springs. If on first trying a plate in the mouth it is a perfect adaptation, he thinks there is something wrong; that it is another trick of that unreliable rubber.

Dr. Hayes thinks that the plate springs after the scraping and paring off of one side in finishing. This outside surface so removed has after the mechanical pressure, acted as the counterpoise against springing.

Dr. Whitney has demonstrated that rubber plates will spring. Cited instances where after finishing and laying away, the plate sprung sufficiently to crack gum sections.

Dr. Bristol finds that there is a change in rubber plates after finishing. For instance, we know that the longer rubber plates are worn in the mouth the harder they become.

Dr. Southwick says there are no rules to gauge the contraction or expansion of rubber. It is irregular and uncontrolable in these phenomena.

Dr. Barrett says that here is another chance for him to air his favorite theory of the polarity of the ultimate atoms of inorganic matter. Caloric changes their arrangement by overcoming cohesive attraction. After the radiation of the surplus caloric, cohesion again begins gradually to bring the atoms into closer relationship with each other, and they slowly assume their normal arrangement. Thinks there is the same phenomena in gold or silver plates, but to an inappreciable extent. Thinks that all inorganic matter everywhere, is changing the arrangement of its particles with the absorption or radiation of caloric. That the regular arrangement of the particles of matter around their axis, is a general law, but that according to magnetic changes, or alterations of temperature, the polarity of the original atoms is changed. Uses the term polarity for want of a better one, but without special reference to any electrical or magnetic state.

Dr. Daboll thinks that much of the difficulty complained of may arise from overheating in finishing up.

Dr. McGill has tried in the mouth plates not vulcanized hard enough, and found a perfect adaptation. After finishing the vulcanizing, he found that they did not fit at all. This leads him to think that rubber does change in some way after the vulcanizing. The harder the piece is vulcanized, the greater the amount of warping; therefore he leaves his plates as soft as possible.

Dr. Hayes was invited to exhibit his self-packer, and complied by showing two machines, very simple in their construction and which experiments had shown were well calculated to do their work in a satisfactory manner. He had found, that with heat and steady pressure, rubber gum would freely exude through an orifice between 212° and 250°, but above this latter temperature it seemed to begin to harden and was no longer plastic. His principle was to apply the pressure gradually with the heat above 212°.

Dr. Whitney finds that there is a great multiplicity of the makers of rubber gum. It was a wrong idea for Dentists to seek for light colored rubber. The lighter the color, the more it contains of vermilion, and the softer it is. An excess of vermilion is irritating to the mucous membrane. The softer rubber gum is, the less will be the strength after vulcanizing; therefore the darker and harder the rubber, the better. Pure rubber and sulphur is always dark in color.

Dr. French has had patients who could not use the rubber as ordinarily manufactured, owing to its irritating the mucous membrane. Used pure rubber without any coloring matter with success, and found it much more tough and elastic.

Dr. Southwick was now invited to give a mechanical clinic. He demonstrated the making and tempering of excavators and other operating implements.

### WEDNESDAY P. M. SESSION.

The Society on being called to order by the President proceeded to designate by ballot the place for holding the next semi-annual meeting. Buffalo was on the first ballot selected. Time, the first Tuesday in May, 1868.

The next subject for discussion was on Materia Medica, and its relations to Dental Practice. Dr. Bristol, essayist.

Dr. Snow understands this subject, as meaning the application of general remedies for the prevention or healing of diseases of the teeth. Thinks Dental caries is very much owing to vicious secretions of the mouth. To correct this, would try to raise the general tone of the system, and prescribe alkaline washes for the mouth.

Dr. Daboll condemns the indiscriminate prescribing of preparations of iron by physicians. It should not be given where anything else can be safely substituted, as it has such a strong affinity for the salts in the teeth.

Dr. Whitney,—this is one of the most important subjects in connection with Dentistry. Would correct vicious secretions by a change of diet and habits. Would also use some form of mercury as an alterative. Condemns the muriatic tincture of iron as injurious to the teeth, dissolving and softening them. Has recommended patients whose physician had prescribed this for them, to use lime water or a solution of Sup. Carb. Soda as a mouth wash, and to use the tooth brush freely after taking the mixture. In neuralgia, and forms of tic doloreaux, as these are only symptomatic, would recommend constitutional treatment.

Dr. Lynch objects to formulas being given for Dentists to follow. Thinks that if we are not competent to make our own diagnosis, we had better turn the case over to a regular physician. Thinks that until we thoroughly understand Materia Medica, we had better not meddle with things out of our province.

Dr. Whitney remarked that when he had a patient with an offensive breath from catarrhal or other affections, insisted upon their gargling the mouth with a solution of chlorate of sodium.

After resolutions of thanks to Drs. J. L. Walter and L. W. Bristol for generous hospitality, the Association adjourned to meet at Buffalo on the first Tuesday of May, 1868.

